# The Institutional Worker

**Published:** 1918-06-29

**Authors:** 


### THE INSTITUTIONAL WORKER

**Being a Special Supplement to "The Hospital"**

### OUR BUREAU OF INFORMATION.

### Rules for Correspondents.

1. Every letter must be accompanied by the coupon to be cut from the back cover (inside page) of The Hospital, current issue, and must contain the name and address of the correspondent with pseudonym for publication if desired. Replies by post cannot be given save under exceptional circumstances at the Editor's discretion.

2. Letters from Approved Homes in reply to special needs published in the Bureau should state terms and full particulars, and be sent prepaid under cover to the Editor of the Bureau with name written across coupon for identification.

3. Proprietors of Homes which have not yet been entered on the List of Approved Homes, but have spare accommodation likely to suit special needs, are invited to write for an application form for registration. The fee for registration, which includes two announcements of the Home in the Bureau and other privileges, is 10s.

4. All communications to be addressed to the Editor of The Hospital, 28 Southampton Street, Strand, London, W.C. 2, and marked "Bureau of Information."

### INSTITUTION FACTS AND FIGURES.

### The Organisation of a Creche in a Munition Factory.

**Answer to May Question.**

**By J. S.**

The question was:

**A large firm employing about 2,000 women workers, many of whom are married, are anxious to start a crèche or day-nursery, for the benefit of the married women, who will be enabled to leave their babies there during working hours. Describe fully the equipment and accommodation required for about fifty babies, and the estimated cost for one year's running. There is already a good welfare department—with two welfare supervisors and two nurses—and it is estimated that the latter could help for a short time each day, and the supervision of the creche would come directly from the head welfare worker.**

A day nursery receiving fifty children should have two nurseries—one for older children, and the other for the small babies. These nurseries may be covered with cork carpet, and should be at least 36 ft. by 20 ft. by 14 ft. each. That for the older children should contain three low tables and benches with back-rests or chairs to accommodate thirty-six children for meals; a thermometer and clock, a chest of toys, and thirty stretchers, piled in a corner when not in use. Each stretcher should have a pillow and blanket. There should be easy access to the garden, which may be asphalted. The large nursery for the babies should contain fourteen to twenty cots, each fitted with mattress, under-blanket, mackintosh, pillow and pillow-case, also one or two top blankets. If the nursery has an open-air glass-covered extension, with canvas side-curtains, the cots can remain out of doors, except in the coldest weather, and the older children can sleep here on their stretchers; otherwise the cots should be moved into the garden whenever possible. Both nurseries should have two slow-combustion stoves with fire-guards.

There should be two bathing-rooms, fitted with large slop-sinks or baths (the former are most convenient) with hot and cold water laid on. In one of these rooms, if large, the children can be received, bathed, and dressed in the crèche clothes. The clothes required for this purpose are:—Fifty jerseys, thirty-six knickers, fifty slips, fifty bibs, socks, and six dozen napkins for the babies. These garments should be arranged neatly on shelves or in cupboards. After the children are bathed and dried on two large trestle tables covered with long bath towels or sheets, their own outer garments are put into numbered bags and hung on pegs, or in wire baskets, of which there should be fifty, one for each child.

There should be an isolation-room or bungalow for cases of illness; also a milk-room (laboratory), where milk mixtures are prepared, having basin with hot and cold water, gas-ring, shelves or cupboard, large bowl (enamel or glass) for bottles, teats and valves, milk saucepan, bottle-brushes, etc.; a dining-room for the staff; an office and committee-room, where the register, enrolment-book, housekeeping account book, and diet-sheets, etc., are kept; a kitchen, scullery, pantry, with crockery for children (thirty-six mugs, thirty-six plates, thirty-six spoons), and larder; a large drying yard with pram-shed and laundry. A mangle and ironing-table will be needed.

The first floor is usually devoted to the nurses—bedrooms, bathroom, lavatory, etc.

Under the conditions mentioned the resident staff might consist of matron, one hospital-trained nurse, and two or three probationers, also a good cook-general. The nurses should be responsible for keeping their bedrooms and the nurseries clean and tidy and laying and removing their meals; the roughest work to be done by the servant. Towels and napkins should be washed, wrung, and hung out twice daily. Slips and pillow-cases must be washed twice weekly.

The following would be the cost of running such a crèche:—

| | | Per Year £ |
|---|---|---|
| Staff salaries ... ... ... | | |
| Matron ... ... | £45 | |
| Nurse ... ... ... | £20 | |
| 2 Probationers (£10 each), | £20 | |
| Cook-General ... | £20 | 105 |
| Food: Staff ... | £120 | |
| Babies ... ... | £130 | 250 |
| Fire and light ... ... | | £20 |
| Rent, rates, etc. ... ... | | £75 |
| Wear and tear ... ... | | £10 |
| Total ... ... ... | | £460 |

Rations have reduced the cost of living, and where there are a large number to cater for the cost is proportionately smaller.

The chief items of food would be:—Milk, about £30 a year; bread, about £30 a year; meat, about £25 a year; fish, eggs, about £20 a year; groceries, about £120 a year; vegetables, about £15 a year; soap, soda, etc., about £5 a year; chemist's account, about £5 a year. Total, £250 a year.

### JUNE QUESTIONS.

**1. Equipping an X-ray Department.**

Give a list of equipment for an X-Ray, Electrical, and Massage Department in a hospital of 180 beds.

**2. Restrainer for Diphtheria Case.**

Describe a simple, washable restrainer, suitable for helping to keep flat in bed a young child suffering from diphtheria. Such a patient, after the first week or so, does not feel ill, and it is almost impossible to keep a child flat in bed without some kind of restrainer.

### RULES.

The following rules must be observed:—

1. Contributions must be written on one side of the paper. Brevity and terseness are desirable features. The MSS. must bear the name and address of the sender and be accompanied by coupon to be cut from the back cover (inside page) of the current issue of THE HOSPITAL. A pseudonym must be chosen if the name is not to be published.

2. Contributions must be addressed to the Editor of THE HOSPITAL, Institutional Worker Supplement, 28 & 29 Southampton Street, Strand, London, W.C. 2, should reach him before the end of the current month, and be marked in the left-hand corner "Facts and Figures."

**A minimum payment of Five Shillings will be made for each published answer.**

**The Editor will be glad to receive correspondence and to consider contributions upon all subjects relating to institutional work which affect the welfare of institutional workers.**

### ENQUIRIES AND ANSWERS.

*WAR MATTERS.*

**War Work Abroad.**

PROBABLY your friend's best course would be to apply to the Secretary, British Red Cross Society, 83 Pall Mall, S.W. 1, stating her wishes and qualifications.—H. J. W.

*EMPLOYMENT AND TRAINING.*

**Nursing the Sick-Poor.**

YOU no doubt refer to the Ranyard Nurses. Free district training and lectures are given to fully-trained hospital nurses desirous of nursing the sick-poor of London. You must apply to the Secretaries, Ranyard House, 25 Russell Square, W.C.—FREDA.

**Training for Lady Dispenser.**

YOUR friend can qualify to work under a doctor only by passing the Assistants' Examination of the Society of Apothecaries of London. For this six or twelve months is sufficient time to study. The highest degree a woman dispenser can obtain is that of pharmaceutical chemist, but this necessitates a long course of study. If you write to the Secretary, Pharmaceutical Society, 17 Bloomsbury Square, London, W.C., you will obtain all the further information you require.—HEALTH VISITOR.

*MISCELLANEOUS.*

**Reclaiming Dressings.**

THE shortage of dressings and their high price have led to the consideration of the question of reclaiming soiled dressings and using them again. There is, of course, a prejudice against this practice, and in normal times very few would have given a second thought to it. There is no doubt, however, that large quantities of dressings which could be made available for further use are daily destroyed. The top layers of absorbent wool dressings can be made use of without any treatment. Provided gauze is of a reasonable quality it can with advantage be washed and sterilised. A little management of the staff should provide the needful time and avoid increasing labour. The effort required is relatively small; the soiled dressings can be collected in suitable bags and sent to the laundry; there they should be placed in a meshed bag and allowed to soak over-night in cold water. In the morning they should be put into a washer and rinsed in cold water for ten minutes. This should be followed with three-quarters of an hour hot washing with soap and soda; after rinsing, steam should be turned on and the dressings boiled for three-quarters of an hour. They are then ready to put in the hydro-extractor, and they may be sent moist to the dressing-room. —BRIDGET.

**A Useful Crutch Seat.**

WALKING with the aid of crutches, even under the most favourable conditions, is, to say the least of it, tiring. With a view to providing a seat which can be conveniently carried in the patient's pocket, Messrs. Allen and Hanburys, Ltd., of 48 Wigmore Street, W., have placed on the market the "Swarren" crutch seat. The seat consists of stout canvas webbing 3 inches wide, with plated-steel end attachments for affixing to the crutch hand-pieces. There are no screws or complications whatever: the seat automatically locks itself to the crutches with steel clips. The price is 7s. 6d. Crutches with a special locking arrangement for use in conjunction with the seat can also be obtained.—E. W. S.

**Hand-propelled Tricycle.**

WE suggest a hand-propelled invalid tricycle for your disabled friend. It is more suitable than a wheel-chair for the streets. An exceedingly well-designed tricycle of this description is made by B. W. Peters, 36-38 Hatton Garden, London, E.C. 1. It is easily steered by pressure on the back support, is comfortable, and safety is ensured by two powerful brakes. The price is twenty-eight guineas.—TONY.

